# The Emergence of Nanofluidics for Single-Biomolecule Manipulation and Sensing

**DOI:** 10.1021/acs.analchem.4c06684

**Published:** 2025-04-17

**Authors:** Marzia Iarossi, Navneet Chandra Verma, Ivy Bhattacharya, Amit Meller

**Affiliations:** Faculty of Biomedical Engineering, Technion -IIT, Haifa 3200003, Israel

## Abstract

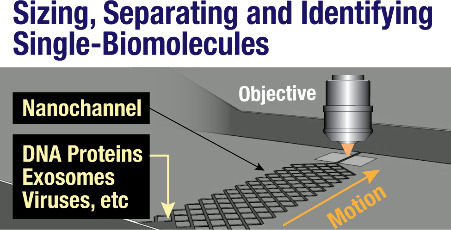

Driven by recent advancements in nanofabrication techniques, single-molecule sensing and manipulations in nanofluidic devices are rapidly evolving. These sophisticated biosensors have already had significant impacts on basic research as well as on applications in molecular diagnostics. The nanoscale dimensions of these devices introduce new physical phenomena by confining the biomolecules in at least one dimension, creating effects such as biopolymer linearization, stretching, and separation by mass that are utilized to enhance the biomolecule sensing resolutions. At the same time, the suppressed diffusional motion allows for better single-molecule SNR (signal-to-noise ratio) sensing over time. In particular, nanofluidic devices based on nanochannels have been established as promising technologies for the linearization of ultralong genomic DNA molecules and for optical genome mapping, opening a window to directly observe and infer genome organization. More recently, nanochannels have shown promising capabilities for single-molecule protein sizing, separation, and identification. Consequently, this technology is attracting remarkable interest for applications in single-molecule proteomics. In this review, we discuss the recent advancements of nanochannel-based technologies, focusing on their applications for single-molecule sensing and the characterization of a wide range of biomolecules.

## Introduction

Advancements in microfluidics have made it possible to perform complex *on-chip* operations, such as the filtration and mixing of biomolecules and the execution of multistep chemical processes using ultrasmall volumes. In the field of molecular diagnostics, they have led to the realization of devices capable of detecting biomarkers in low concentrations from physiological fluids.^1−4^ Recent progress of nanofabrication technologies have enabled the development of *nanofluidics*-based platforms capable of sensing and manipulating single molecules, effectively defining the ultimate next-generation of single-molecule diagnostic devices.^5,6^ In these nanofluidic platforms, at least one characteristic length scale spans the range of a few to several hundreds of nanometers. As a result of the large surface-to-volume ratio and the high degree of confinement of molecules in spaces that are often their own size, unique and unusual phenomena arise that are typically not observed at the microscale.^7−9^ For instance, access to transport properties at the nanoscale acts as the basis for electrical sensing in nanopores, which are essentially zero-dimensional confinement devices, highly facilitating the development of robust single-molecule sensing and sequencing technologies.^10^ Nowadays, nanopore-based devices play a dominant role in the analysis of DNA, RNA, and proteins at the single-molecule level.^11,12^ Other nanoscale devices that confine molecules in only one or two dimensions have also emerged. Due to the nanometric subwavelength volumes delimited by these nanofluidic devices, nanochannels have been integrated with optical techniques for single-molecule investigations.^13−15^ This has prompted the implementation of nanofluidics in advanced applications such as large-scale optical genome mapping and the identification of individual proteins. The combination of microfluidics and nanofluidics has acted as an additional boost to the development of point-of-care diagnostics and lab-on-a-chip devices..^16,17^

In this review, we provide an overview of the current state-of-the-art nanofluidic devices for biological single-molecule analyses. First, we describe their conceptualization and fabrication, including the devices’ assembly and encapsulation. Then, we discuss how the various types of nanofluidic devices have provided excellent platforms for the manipulation, sorting, and optical mapping of DNA and other polymeric chains. Then, we move to the emerging applications involving the identification of proteins and the characterization of other relevant biomolecules, covering many of the promising devices and optical techniques that have been developed to date. To conclude, we discuss the further optimization that nanochannel-based devices require and envision their potential impacts in the diagnostics and bioanalysis fields.

## Materials, Designs, and Fabrication Approaches

Unlike microchannels, in nanochannel devices, at least one of the dimensions is at the nanoscale, spanning from just a few to several hundreds of nanometers. There are different types of nanochannels, which can be categorized based on their aspect ratio (their height to width ratio).^18^ Specifically, planar nanochannels have a low aspect ratio that can be readily fabricated by standard lithography approaches. These devices have been widely used for single-molecule optical detection. In contrast, vertical nanochannels have a high aspect ratio and are considered to be more difficult to fabricate. The vertical devices have received significant attention for filtration and electrical sensing.^19−23^ In this review, we will mainly focus on planar nanochannels, which involve simple and scalable fabrication approaches while offering capabilities to rescale typical microfluidic operations such as separation, concentration, and filtration, using sub-picoliter volumes or even less than that. At the same time, these devices also achieve high throughput in terms of detection when compared to other single-molecule nanosystems.^24,25^

**Figure 1:**
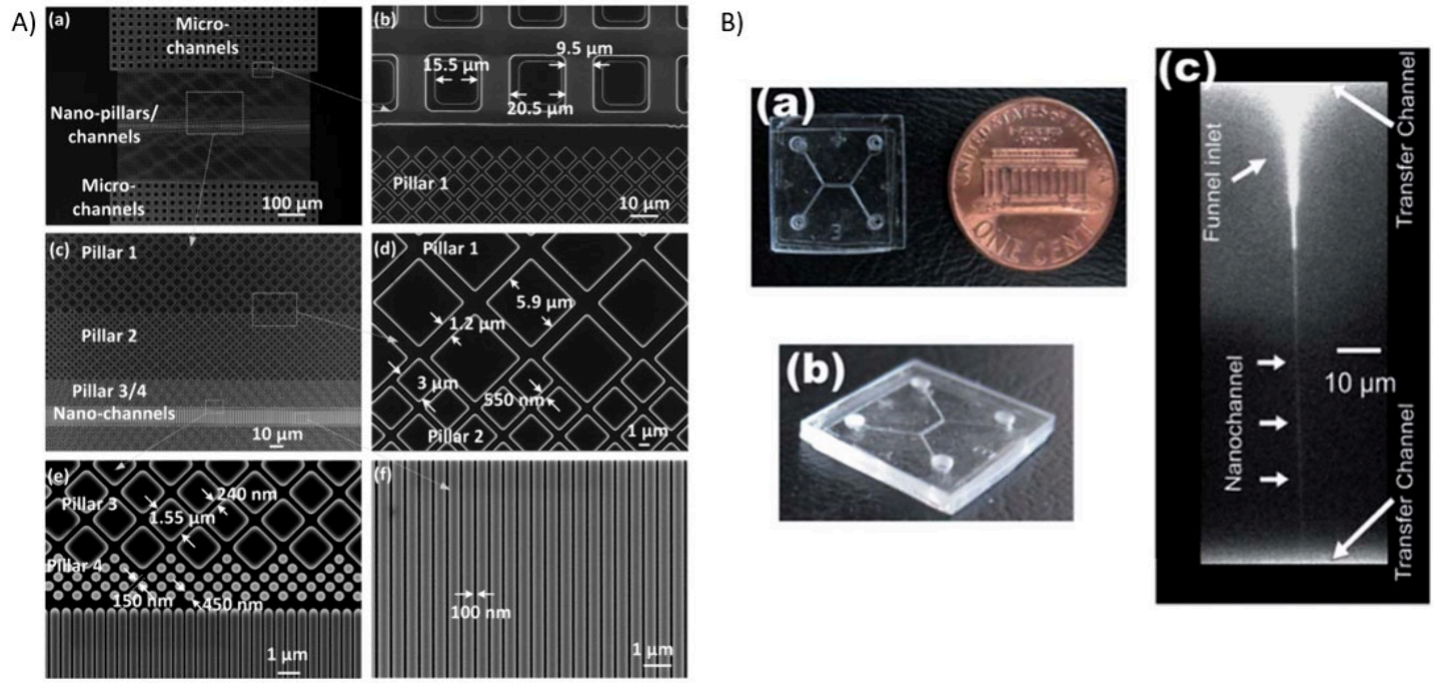
Examples of fabricated nanofluidic devices. (A) Scanning electron microscope (SEM) images of micro- and nanofluidic structures fabricated with deep-UV (DUV) lithography. The sequence of images from low to high magnification highlights the connection of microchannels to nanofluidic channels and diamond-shaped nanopillars. Reproduced from ref (29). Copyright 2015 American Chemical Society. (B) On the left is another example of a nanofluidic device made of PMMA after thermal fusion bonding with a PMMA coverslip, and on the right is a fluorescence image of a single nanochannel filled with 0.1 mM FITC to show its geometrical features. Reproduced from ref (45). Copyright 2011 Royal Society of Chemistry.

Silicon wafers are widely used as supporting substrates for the fabrication of planar nanochannels that are patterned on a given layer by means of well-established top-down microfabrication techniques, including UV lithography and reactive ion etching.^19,20,26^ The design of the nanochannels and their integration with other nanostructures made by other top-down fabrication tools (Figure 1A) determine the final device functionalities and capabilities in terms of molecular confinement at the nanoscale, fluidics integration with inlet/outlet ports, selective transport, and capture and/or preconcentration of molecules.^27−31^ For instance, a molecular sieving structure design called the anisotropic nanofilter array has been developed for continuous-flow separation of DNA and proteins based on either size or charge.^32^ Glass is also widely used as a supporting substrate for nanochannels since it is optically transparent, chemically inert, and easy to functionalize with coatings made of silane, polyethylene glycol, or other passivation layers.

The assembly of chips with nanochannels patterned on conventional substrates, such as silicon, glass and fused silica, consists of the bonding of the patterned substrate with a glass cover slide.^33−35^ If the substrate is made of glass, glass-to-glass bonding can be achieved by thermal fusion by placing the substrate and the cover slide in contact at temperatures higher than 500 °C, and eventually under a certain pressure. If the chip is made of silicon or metallic materials, a borosilicate coverslip can be typically sealed by anodic bonding by applying an electric potential of several hundred volts between them at high temperatures above 300 °C. In any case, both surfaces need to be as flat as possible with a surface roughness of less than 5 nm and must be thoroughly cleaned, for example, by immersion in a hot piranha solution (3:1 mixture of concentrated sulfuric acid with hydrogen peroxide) and subsequent air or O_2_ plasma treatment before the bonding procedure.^36^ Additionally, bonding techniques have been developed to bond glass coverslips and flexible glass sheets under high pressure and low temperature (100 °C).^37^ After bonding, the flexible glass can be reversibly deformed under pressure, forming a valve that controls the fluidic transport in a certain area connected to a network of channels.

One limitation of conventional top-down fabrication techniques of nanofluidic devices is that they are typically time-consuming and expensive, limiting their industrial scale-up. For this reason, alternative strategies based on nanoimprint lithography have been developed.^38−40^ Here, a thermoplastic is embedded with a mold under pressure at a temperature above the glass transition, then polymer bonding techniques are employed for the encapsulation of the device. Although elastomeric materials, such as polydimethylsiloxane (PDMS), are often not robust enough at the nanoscale, optimizations of the polymer formulation have improved the mechanical properties to the point that nanochannels with tunable features can be fabricated.^41−44^ Similarly, thermoplastics such as poly(methyl methacrylate) (PMMA), polycarbonate (PC), cyclic olefin copolymer (COC) and polyethylene terephthalate (PET) have also been explored as materials for nanofluidics applications (Figure 1B).^45,46^

## Nanofluidic Devices for Genomic Analyses

Nanofluidic platforms based on nanochannels have become a central tool in the field of optical genome mapping due to two main features: (i) their unique capabilities to linearize individual DNA molecules and other polymeric chains through physical confinement and (ii) their integration with genetic barcoding routines, namely sequence-specific labeling, supported by optical sensing through multicolor fluorescence single-molecule imaging.^47,48^ Remarkably, specific designs of nanochannels for optical mapping have been translated to a commercialized product, such as nanofluidic devices consisting of an array of nanochannels with a cross-section of 36 × 36 nm^2^ from BioNanoGenomics.^49^

### Delivery and Linearization of DNA Molecules Confined in Nanochannels

**Figure 2:**
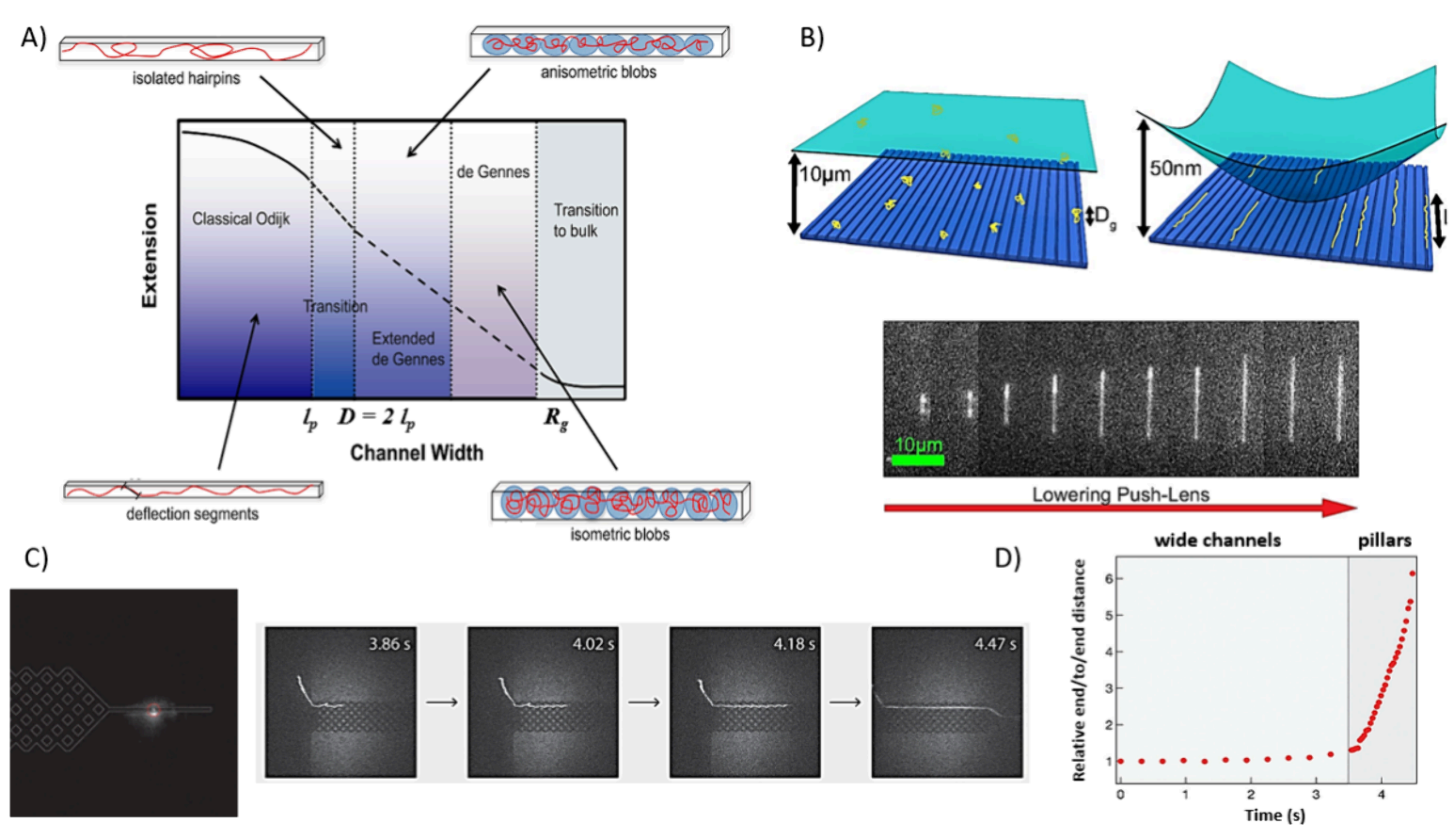
DNA confinement and linearization in nanochannels. (A) Schematic overview of the physical regimes in nanochannel confinement (*l*_p_: persistence length, *R*_g_: radius of gyration). Reproduced from ref (54). Copyright 2012 IOP Publishing Ltd. (B) At the top: scheme of the DNA-loading procedure with the push-lens mechanism. When the chamber height has microscale vertical dimensions, DNA molecules are coiled conformations, while when the push-lens is lowered DNA molecules align in the nanochannels due to the increasing physical confinement. At the bottom sequence of frames for a λ-DNA molecule extending in a nanochannel of 27 nm, the push-lens is lowered. Reproduced with permission from ref (57). (C) Image of a nanofluidic device integrating a nanopillar array with a solid-state nanopore. Sequential optical images of an extremely long labeled genomic DNA (∼400 kbp) extracted from human cell-lines, being linearized while moving into the nanopillars acting as physical barrier, and being delivered to the nanopore for electrical sensing. (D) End-to-end distance of the DNA molecules relative to its starting end-to-end distance in the channel as a function of time. Reproduced from refs (74). Copyright 2019 American Chemical Society.

Several theoretical approaches have been developed to describe how a DNA molecule elongates along the nanochannel depending on the degree of physical confinement and eventually in the presence of an electrostatic field gradient.^50−52^ The key physical parameters involved in these models are the effective width *w* of the DNA backbone in a buffer solution, its total contour length *L*, and its persistence length *l*_*p*_ (∼54 nm for dsDNA). To describe the case of a DNA molecule confined in a square nanochannel of size *D*, another characteristic length must be introduced to take into account the interaction of the DNA molecule with the channel’s walls. This is called depletion length δ, so that the effective length of the channel is *D*_eff_ ≈ *D* – δ. The strong confinement regime, also known as the Odijk regime, is characterized by the fact that *D ≪ l*_p_, and it is possible to show that the DNA stretching increases as the size of the channel *D* decreases (Figure 2A). Several reviews in the literature describe in detail how DNA molecules stretch in nanochannels from the theoretical point of view.^50,53,54^

Another advantage of nanochannel-based devices is that an external electrophoretic force can be added to the system to facilitate DNA threading and remeasuring of its motion by reversing the field.^55^ It has been demonstrated that to obtain the maximum elongation of DNA in a nanochannel, not only the geometry of the channel but also the ionic strength of the solution in the channels, which affects the molecules interactions with the channel walls, need to be optimized.^56^ To transform a macroscale flow cell into a nanofluidic device without the need for permanent direct bonding, a curved surface of a convex lens can be used to deform a flexible coverslip patterned with nanochannels, achieving high stretching (90%) of DNA molecules (Figure 2b).^57^ During DNA linearization, as the geometrical features of the channel decrease from the microscale to the nanoscale, for example, by progressively narrowing elastomeric channels, the electrophoretic force acting on the DNA must overcome a significantly higher entropic barrier to allow it to enter the confined space of the channel itself.^58−61^ To overcome this aspect, funnel-shape nanochannels have been designed to gradually increase the degree of confinement of the molecules and decrease the entropic barrier to the point that the electric field threshold required to pull the DNA molecules toward the entrance of the nanochannel can be reduced up to a factor 30.^62^ Inlet structures with different shapes connecting microfluidic channels to nanochannels, including patterned pillar arrays, grooved, V-grooved, and funnels, have been tested to understand which design is able to better enhance the capture rate of DNA molecules.^63^ Indeed, funnel-shaped channels have shown better capabilities to enhance the capture rate of DNA compared to the other mentioned designs.^64^ Additionally, by designing an asymmetric lattice of crossed nanochannels with diameters of 80–140 nm, Riehn et al. observed a preferred direction for DNA orientation and transport under direct current electrophoresis.^65^ The preferred axis of orientation and transport is switched by 90° by applying an ac voltage.

Importantly, nanochannel-based devices can be used not only to study fully linearized DNA but also to investigate topological events involving knot formation and the interactions between DNA knots along the chain, as they are known to play an important role in transcription and other cellular processes as a function of the degree of confinement.^66−69^ From a computational study of a system consisting of a nanochannel and a confined DNA molecule containing two knots, it has been shown that the knots tend to stay separated, implying that their intertwined state is inhibited because the probability that the two knots interact with each other becomes very low under a strong confinement regime.^70^ On the other hand, for wider channels under a weak confinement regime, namely when *l*_p_ < *D*, the two knots can interact with each other forming a larger intertwined knot, meaning that even more complex polymeric chains can in principle be studied by tailoring the degree of confinement.

For applications involving ultralong genomic DNA, interfacing these molecules with nanochannels can be challenging because of the high entropic barrier and the higher probability of clogging the channel with long and coiled DNA. To this end, an array of microfabricated pillars acting as physical barriers provides a strategy to fully stretch genomic DNA by gradually increasing the confinement through the pillar’s arrangement and thus reducing the entropic term related to the DNA capture and unfolding.^39,71,72^ By tuning the geometry of the pillar array, such as the pillar diameter, the reciprocal distance, and the displacement angle, DNA of different lengths, namely 166 and 48.5 kbp, have been stretched and sorted.^73^ Furthermore, a nanofluidic platform supporting an array of pillars can be integrated with another single-molecule sensing device consisting of a solid-state nanopore as a functional element to sort, stretch, and deliver extremely long intact genomic DNA molecules (∼400 kbp), extracted from a cancerous cell line and labeled with YOYO-1, directly to the entrance of the nanopore.^74^ The migration of the DNA through the array of pillars is driven by a hydrostatic pressure gradient and is monitored by fluorescence imaging. When molecules reach, the nanopore they are actively threaded using a strong perpendicularly oriented electrical field, and their translocation process through the nanopore is monitored via the electrical current (Figure 2c). Due to the high confinement of DNA molecules across the array of pillars, the translocation through the nanopore is significantly slowed by more than 3 orders of magnitude compared to the time taken in the absence of the pillar array. This confirms that nanochannels and physical barriers like pillar arrays significantly slow down the motion of DNA molecules and linearize their backbone (Figure 2d), providing a powerful tool for DNA manipulation and for upstream analysis in combination with other sensing platforms.

Sticking and clogging of nanochannels due to the strong interactions of the long, confined DNA with the channels’ walls has been an ongoing issue. In this regard 2D materials, such as graphene, have been proposed as a novel class of materials capable of “self-cleaning” due to their ultrasmooth and clean surface that prevents frictional interactions between DNA and the confining surfaces.^75^ In a recent paper by Cui et al., nanochannels with heights as low as 4.3 nm have been produced on graphene layers using femtosecond laser etching and transferred onto a SiN_*x*_ supporting substrate by a wet transfer approach.^76^ These channels were fabricated with heights of a few nanometers, 30 nm widths, and length-to-height ratios up to 10^3^. Nanochannels on graphene layers have been used to transport, stretch, and analyze DNA molecules of different lengths from 0.2 to 48.5 kbps at different voltages by monitoring the ionic current across the channel and proving the existence of a linear relationship between the residence time and the DNA length. In fact, nanochannels of different materials have been integrated into horizontal two-nanopore systems to detect molecules at the single-molecule level by measuring the travel time across the two entrances from the current blockages over time.^77−80^ Nevertheless, electrical sensing with nanochannels is limited by the spatial sensitivity given by their geometry and therefore ultrathin nanopore-based technologies may provide better signal-to-noise ratio with single-base-pair resolution.^81,82^

### Optical Genome Mapping by Multicolor Single-Molecule Imaging

**Figure 3:**
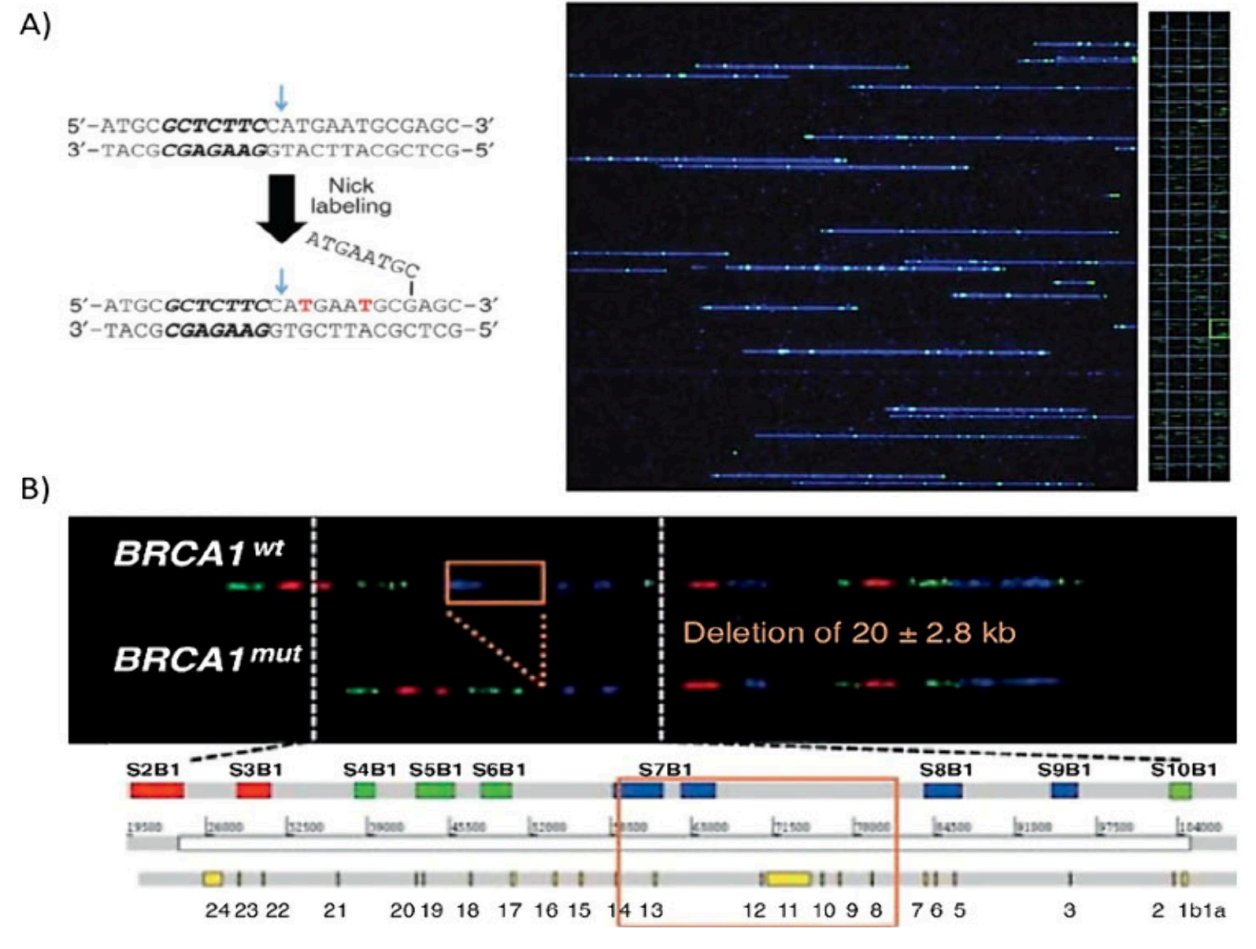
Genomic DNA labeling strategies for nanochannel-assisted optical mapping. (A) For the labeling, a nicking endonuclease is used to introduce single-strand nicks in the double-stranded DNA at specific sequence motifs. Then, fluorescent dye-conjugated nucleotides were incorporated at these sites by Vent (exo-)polymerase. On the right is an image of a single field of view (FOV 73 × 73 μm) containing a mixture of nick-labeled DNA molecules in the nanoarray. This FOV is part of the 108 FOVs shown in the bottom part of the panel (outlined in green). Each FOV can accommodate up to 250 kb of a DNA molecule from the top to bottom. Reprinted with permission from ref (84). Copyright 2012. (B) Example of known BRCA1 large rearrangements detected in breast cancer patients. Specifically, deletion of blue signal S7B1, including a large genomic portion between signals S7B1 and S8B1, is observed. Reproduced from ref (96). Copyright 2012 Wiley.

Other than the linearization of biopolymers, nanochannels are excellent nanofluidic platforms for single-molecule sensing by means of optical techniques, such as fluorescence imaging. The biopolymer confinement in nanochannels largely suppresses the thermal fluctuations of the molecules, hence facilitating high SNR (signal-to-noise ratio) optical measurements.^83^ In particular, nanochannels are ideally suited for genomic applications of long DNA, where the molecules can be driven in and out of the channel multiple times to perform multiple rereads of the same molecule while linearizing them during the motion through the channel. By combining labeling approaches of specific sequence motifs of DNA and single-molecule imaging of the labeled DNA in nanochannel arrays, it is possible to construct maps of the physical distances between occurrences of the sequence motifs.^84−86^ A plethora of labeling strategies for DNA barcoding have been established to mark specific sequence sites with organic dyes.^87,88^ For instance, to achieve site-specific fluorophore tagging of DNA, a nickase-based strategy to target the specific 20 bases across the whole genome has been developed. Specifically, the Cas9 enzyme creates a DNA nick according to a guide RNA and then fluorescent nucleotides are incorporated nearby the nicking sites.^89^ Multicolor labeling is obtained by combining the Cas9 sequence-specific labeling with other labeling techniques of specific sequence motifs, for instance, with the direct label enzyme (DLE-1) approach.^90^ In Figure 3a, an example of selective labeling is reported by introducing a nicking endonuclease to produce single-strand nicks in the double-stranded DNA and then attaching fluorescent dye-conjugated nucleotides to these sites by Vent (exo-)polymerase.^84^ On the other side, enzyme-free labeling approaches have also been used. These are based on the competitive binding of YOYO-1 and netropsin to highlight the DNA contour and, at the same time, create a continuous sequence-specific pattern based on the AT/GC variation along the DNA.^91^ The integration of nanochannels with multicolor imaging has been achieved for the detection of DNA and histones in individual chromatin fragments at about 10 Mbp/min, enabling epigenetic analysis through the identification of DNA methylation on individual molecules.^92^

An important aspect of optical genome mapping is that the degree of confinement of DNA in the nanochannel affects the magnitude of thermal fluctuations. This, in turn limits the capability to resolve two adjacent fluorophores in labeled DNA molecules. In optical mapping, nanochannels working under the Odijk regime show better genomic resolution because (i) the DNA is fully stretched, preventing partial folding, loop formation, etc., and (ii) the thermal fluctuations are minimized. Furthermore, the latter can be experimentally measured and then removed from the overall map by collecting images at short exposure times (i.e., 40 ms) since adjacent fluorophores on the DNA act as fluctuation reporters of the collective thermal motion of the molecule.^93,94^ By analyzing the optical genome maps of 154 individuals from 26 populations, it was possible to characterize the phylogenetic population patterns of large structural variants in the human genome.^95^ Similarly, optical genome maps have been collected and compared to genome references, often in combination, to study DNA replication, damaging mechanisms, compaction, and chromosomal rearrangements (for instance, see the example reported in Figure 3b).^96−105^ By combining the analysis of optical genome maps and short-read sequencing, it is possible to recover information on interspersed sequences that do not align with the reference genome, as achieved in a study focusing on the optical genome maps obtained from several cancer cell lines, thus highlighting the potential of genome mapping in cancer research and other diseases.^106−112^ Among other recent applications, optical DNA mapping has been used to characterize plasmids carrying the mcr-1 gene, which has been identified to be responsible for resistance to colistin, a last-resort antibiotic for Gram-negative infections.^113^ In this study, plasmids isolated from patient samples were treated with CRISPR-Cas9 to target the specific region containing the antibiotic resistance gene of interest. Then, DNA molecules were labeled with a single-step YOYO-netropsin labeling strategy, loaded, and driven by pressure into an array of parallel nanochannels. Each channel was imaged by fluorescence microscopy to extract the intensity versus length plot from each individual plasmid with the aim of identifying the location of the resistance gene from the intensity profile. Therefore, the barcodes obtained by optical mapping and those generated by nanopore sequencing were compared for two isolates T4F_1 and T6F_1, which carry the mcr-1 gene in 116 and 68 kb-long plasmids, respectively. The match between the barcodes obtained with optical mapping and nanopore sequencing was found to be very good (*P* < 0.01) for these specific isolates, which were located on relatively large plasmids, but also acceptable for other six isolates located in short plasmids of about 30 kbp, which were more challenging to characterize by optical DNA mapping due to the limited resolution imposed by the thermal fluctuations, incomplete linearization and other factors.

Alternatively, optical DNA mapping with nanochannels has been used for bacterial phenotyping by analyzing individual DNA molecules extracted via plug-lysis from bacteria in patient samples. The aim was to identify *Escherichia coli* and *Klebsiella pneumoniae* at the strain level through the matching of each individual intensity profile against a reference database.^114^ Recently, 47 different plasmids encoding the *bla*_CTX-M_ genes have been characterized and grouped by optical genome mapping in a nanochannel-based platform to potentially elucidate their role in the evolution of bacteria that produce extended spectrum beta-lactamases (ESBL). They were isolated from *E. coli* (*ESBL-E. coli*) strains during the early stage of the ESBL pandemic of 2009–2014 in western Sweden.^115^ In this way, two plasmids are similar if their barcodes can be considered similar by using the *p*-value principle, that is, if a value of 0.01 or lower is obtained. If three or more barcodes are similar according to the *p*-value principle, the corresponding plasmids are assigned to the same group, and indeed a large number of the investigated plasmids were classified into 5 different groups. These findings could shed light on the relevance of some of these plasmids during the ESBL pandemic, as they were already circulating at the early stages when antibiotic resistance was still low.

Nanofluidic channels can also be used to study protein–DNA interactions by analyzing DNA–protein complexes.^105,116−118^ For example, two arrays of parallel nanochannels in a perpendicular configuration were designed to load the molecules of interest through the array of channels in one direction, while those in the perpendicular directions were used to exchange buffers. This device was tested by inserting labeled protamine to study the compaction of DNA by protamine, as well as to study the unpacking of precompacted DNA through an increase in the concentration of salt.^119^ In another work about DNA repairing pathways, Möller et al. investigated how two different proteins, the human MRE11-RAD50-NBS1 (MRN) complex and Mre11-Rad50-Xrs2 (MRX) in yeast, are involved in repairing long DNA by promoting DNA bridging.^120^ The two types of bridging events that occur when DNA molecules hybridize are intermolecular bridging, where two or more DNA molecules hybridize together forming concatemers with *n*-times the length of a single DNA molecule, and intramolecular bridging, where a single DNA molecule hybridizes closing its ends into a circle. By measuring the extension of thousands of DNA molecules, it is possible to understand which repair pathway is preferentially promoted by MRX and MRN. In turn, these two proteins demonstrated two different repair pathways, as on average NBS1 induces the formation of circularized DNA molecules, whereas almost solely concatemers are formed in the presence of Xrs2. Finally, parallel nanocavities that can be opened and closed in situ were recently employed to study the spatial distribution and dynamics of plasmids interacting with double stranded DNA as a function of the plasmid number.^121^

## Detecting Individual Proteins under the Diffusion Regime

**Figure 4:**
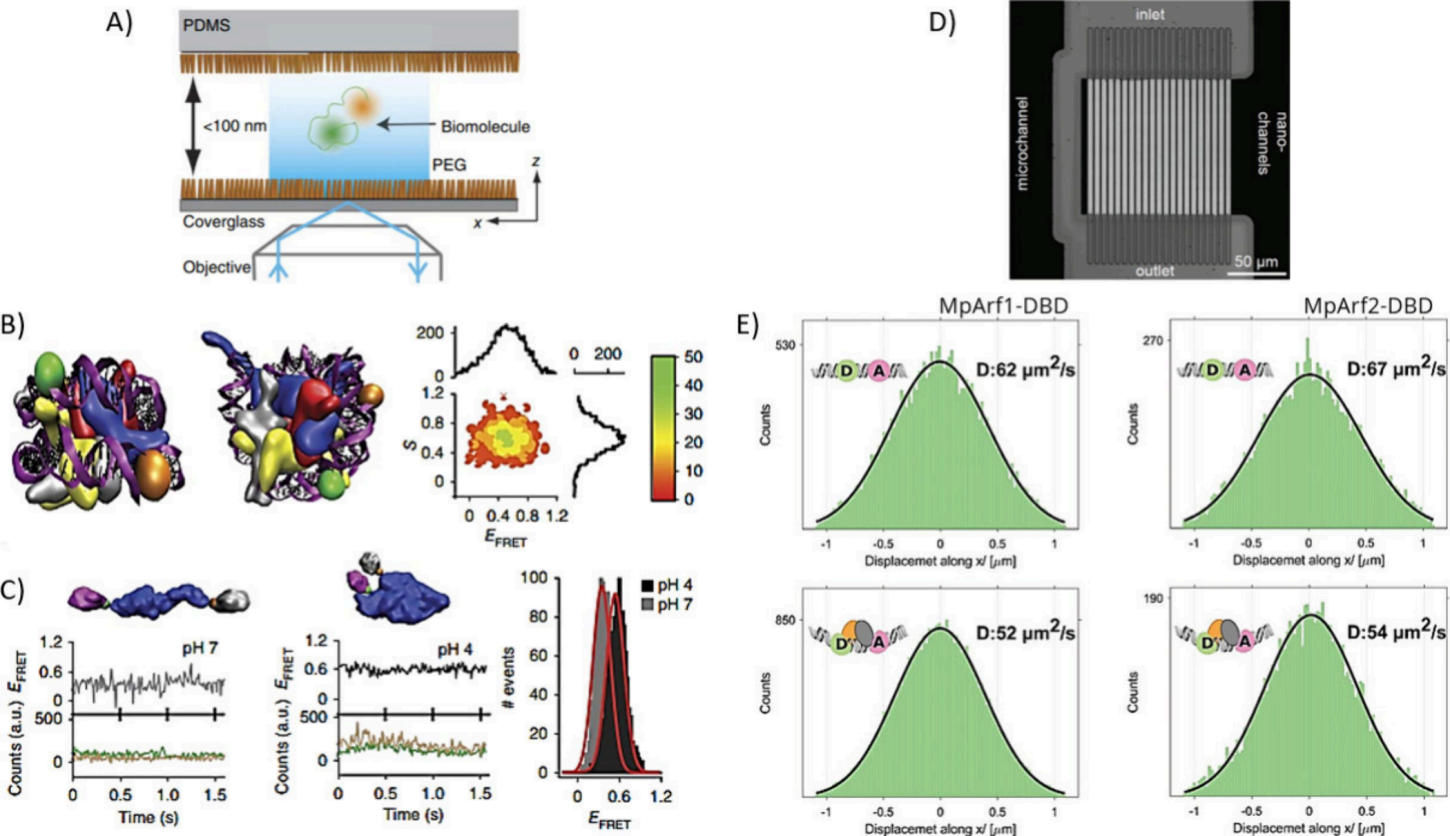
Monitoring conformational changes and binding events through single-molecule FRET in nanochannels. (A) Sketch of a PEGylated PDMS nanochannel with a depth of 100 nm used to image the diffusion of dually labeled molecules at the single-molecule level. (B) Nucleosomes in two orthogonal orientations showing the locations of Alexa 488 (green bead) and Alexa 594 (brown bead) attached to the DNA template (purple). On the right, the histogram of stoichiometry (S) versus FRET efficiency (EFRET) of nucleosomes imaged in the nanochannel-based device using alternating laser excitation is shown. (C) Intensity plot for donor- and acceptor-labeled hTG2 (green and brown, respectively). FRET is shown for pH 7 with *E*_FRET_ = 0.35 (left) and higher FRET is shown at pH 4 with *E*_FRET_ = 0.55 (center) since hTG2 switches from the closed to open conformations by increasing the pH. Reprinted by permission from ref (125). Copyright 2014. (D) Image of the fluidic device with a nanochannel array 200 nm wide in contact with a microchannel that helps to distribute the flow among them due to lower hydraulic resistance. (E) Displacement distribution of the labeled DNA without or with MpARF1-DBD (left) and MpARF2-DBD (right). Reproduced from ref (126). Copyright 2021 Wiley.

The combination of single-molecule optical techniques and nanofluidic devices has provided a powerful approach for the characterization of conformational changes and the size profiling of individual biomolecules. Among other single-molecule optical techniques, single-molecule FRET has recently enabled protein fingerprinting to map the location of individual amino acids and post-translational modifications within single full-length protein molecules, but this approach still requires the immobilization of the labeled molecule of interest to the surface of a cover slide.^122^ Even other optical techniques that are label-free, such as total internal reflection-based evanescent scattering microscopy, require the functionalization of a glass slide with *N*-hydroxysuccinimide (NHS) groups to increase the binding rate or the use of antibodies.^123,124^ However, the reproducibility and efficiency of the collected signals can be hampered by the surface functionalization, since steric and electrostatic interactions can affect the coverage on the substrate and the orientation or position of the fluorescent tags. In addition, single-molecule FRET measurements of diffusing molecules (″burst analysis”) are limited by the short residence time spent by a molecule within the focal volume. The limitations of these techniques can be overcome by the integration of nanofluidics with single-molecule optical techniques. For instance, Tyagi et al. were able to collect the FRET signals of diffusing single molecules within a nanochannel of 100 nm where, due to the high axial confinement, it was possible to collect long trajectories for up to 20 s with the only limitation being the field of view (Figure 4a).^125^ This device was used (i) to study labeled nucleosomes assembled from two copies of four different histones wrapped with a 170-bp dsDNA (Figure 4b) and (ii) to probe the conformational states of human transglutaminase, a 78 kDa four-domain protein, which exists in open and closed states depending on the pH of the solution in which the proteins are dispersed. The conformational changes between the open and closed states were monitored by measuring the variation in the FRET intensity emitted by Alexa 594 and Alexa 488 attached at the C and N termini of the proteins (Figure 4c).

Similarly, in a recent work by Fontana et al., a nanofluidic platform combined with single-molecule FRET has been used to investigate the DNA binding domain of several auxin response factor (ARF) transcription factors and their interaction with the related DNA response element.^126^ In this case, the device consists of an array of nanochannels of 200 nm in contact with a microchannel, where the latter was used to slow down the flow of molecules after their injection with a syringe through a parallel flow control mechanism (Figure 4d). By monitoring the changes in the FRET efficiency and the diffusion coefficient of labeled DNA, the authors were able to monitor the binding of the unlabeled ARF DNA binding domain to donor- and acceptor-labeled DNA oligonucleotides (Figure 4e).

**Figure 5:**
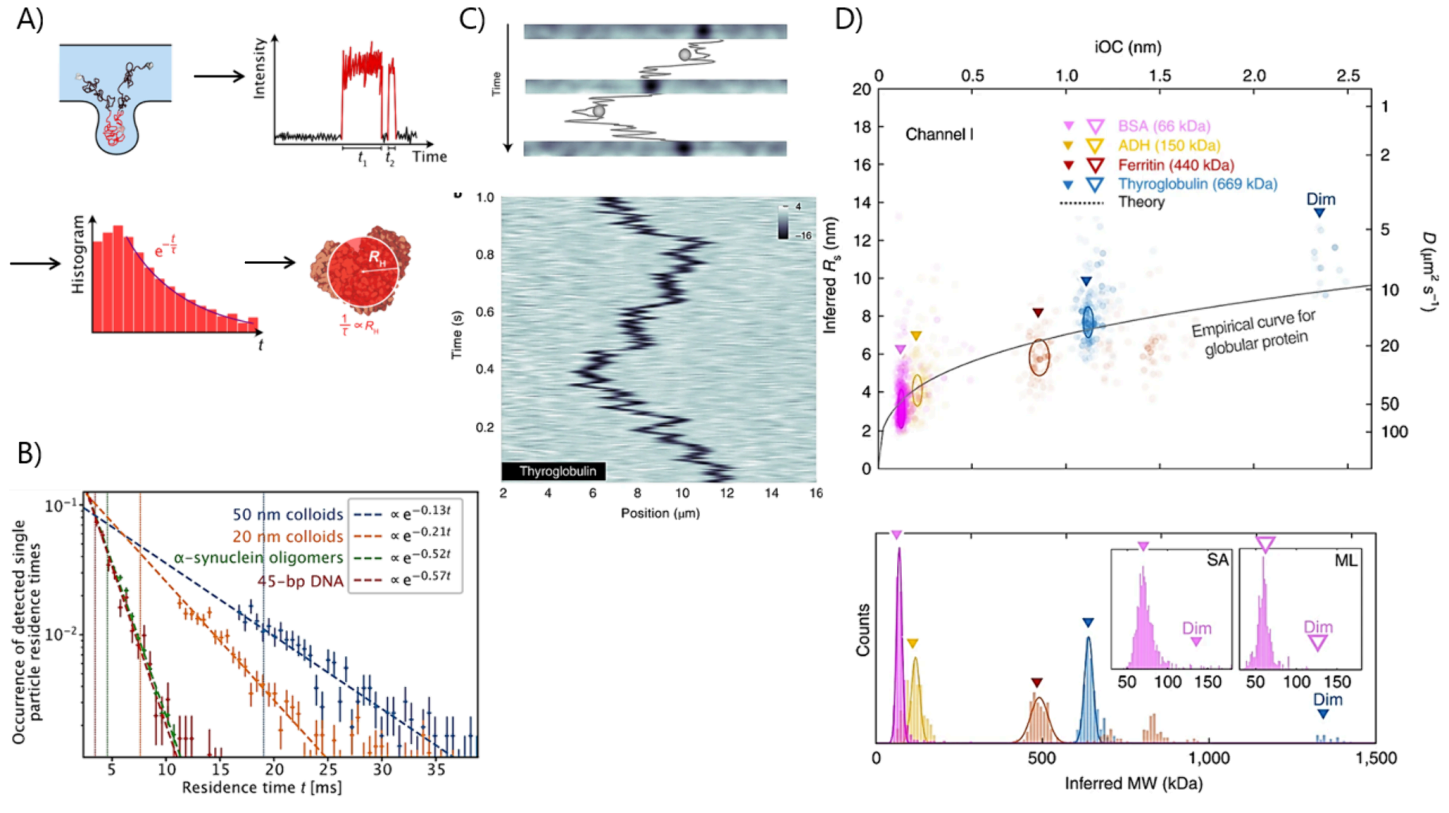
Sensing individual molecules in the diffusion regime with nanofluidic-based devices. (A) In nanoparticle diffusional sizing the molecules are imaged by confocal microscopy while diffusing into and out the nanotrap. From the analysis of the time trace, it is possible to reconstruct the histogram of the residence time and fit it with an exponential function to find the experimental hydrodynamic radius of the molecule. (B) Residence time histogram of different particles obtained with the nanoparticle diffusional sizing approach. Reproduced from ref (128). Copyright 2023 American Chemical Society. (C) Trajectory of thyroglobulin diffusing in nanochannels and imaged by dark-field light-scattering microscopy. (D) Scatter plots of the integrated optical contrast extracted by a particle-tracking algorithm and translated into molecular weight for different proteins. Reprinted by permission from ref (14). Copyright 2022 Springer Nature.

Another example of a nanofluidic platform shows how nanochannels with adjacent nanotraps with dimensions as small as 100 nm in height were fabricated by two photon UV lithography.^127^ The particles diffusing in and out of the nanotraps were imaged by means of fluorescence confocal microscopy and, as a result of the particles’ confinement in the nanotrap, the residence time of the several labeled species, including nanoscale colloids, protein oligomers, and DNA, was increased up to 5-fold within the detection volume of the nanotrap compared to that in free-space diffusion in the adjacent nanochannel. Therefore, by imaging several nanotraps one by one, the time trajectories of a large number of particles were collected, and from those the residence time distribution was obtained. Hence, the size of the particles was extrapolated by the fit of the residence time distribution with an exponential function, representing the decay coefficient inversely proportional to the hydrodynamic radius (Figure 5a, b).^128^ Beyond single-molecule studies, nanocontainers functionalized with polymer brushes can also act as macromolecular gates to control the capture and release of a high number of proteins, up to 1000.^129^ In fact, thermoresponsive polymer brushes locally self-assembled in tiny nanofluidic channels have enabled the active regulation of femtoliter-scale fluid.^130^ In another recent work, a nanofluidic aptamer array was realized by functionalizing a nanochannel supporting nano-in-nano gold nanopatterns with aptamers. This device has been used to capture target proteins at a normal concentration from a sample within an ultrasmall volume by following Poisson statistics and thus forming a single-protein nanoarray.^27^ One advantage of using nanotraps for biomolecule sizing is that it simply relies on the diffusive regime of molecules without any additional external force; however, it requires the scanning of each nanotrap, one at a time, which is a practical limitation for rapid analysis of biomolecules at high throughput as compared to other nanochannel-based devices.

Recently, a new label-free technique named nanofluidic scattering microscopy (NSM) based on the coupling of a nanochannel with dark-field light-scattering microscopy has been established to perform single-molecule imaging in the diffusive regime without the need for labeling techniques (Figure 5c).^14^ The detection principle of NSM is based on subtraction of the scattering intensity produced by an empty nanochannel *I*_*c*_ and the one collected from a nanochannel with a biomolecule inside *I*_t_*= I*_c_ + *I*_m_ – , as the interference term can be significantly larger than the scattering intensity of the molecule *I*_m_ alone. With NSM, it is possible to extract the hydrodynamic radius and the molecular weight of biomolecules, as they can be extrapolated from the optical contrast. This approach has been applied for accurately sizing relatively large proteins larger than 60 kDa (Figure 5d) and extracellular vesicles from a conditioned cell culture medium. Furthermore, NSM has enabled the discrimination of subpopulations of ferritin monomers with different iron contents. In another relevant example of protein characterization in nanofluidic devices, Sasanian et al. used funnel-shaped nanochannels with progressively increasing widths from 300 to 3000 nm to study the physical properties of fluorescence-labeled amyloid fibrils under different degrees of confinement.^131^ As the amyloid’s fibrils stretch in the nanochannels under pressure-driven flow, the variation of their extension extrapolated from the recorded time-lapse movies allows for the measurement of the persistence length as a function of the degrees of confinement. Specifically, the authors focused on the Alzheimer’s related peptide amyloid-β(1–42) and the Parkinson’s related protein α-synuclein, establishing that the former has a higher average persistence length with a broader distribution, though α-synuclein fibrils are on average thicker. Since the heterogeneity of the persistence length does not depend on the average fluorescence intensity per unit length, which is instead related to the number of the filaments and, in turn, also to the fibril thickness, it can be concluded that the stiffness of the fibrils depends on the way the filaments pack more than their number.

### Toward Advanced Applications of Protein Characterization and Identification

Besides the few examples of nanofluidic devices capable of stretching biomolecules and sizing individual proteins in the diffusion regime discussed in the previous paragraph, limitations still exist for the analysis of complex protein samples. Due to the large variety of proteins in each cell, the structural complexity in terms of amino acid (*aa*) composition, the diversity of their three-dimensional structures and the existence of protein isoforms with similar *aa* sequence, protein sensing in nanochannels requires the development of more sophisticated devices capable of probing several intrinsic physical properties simultaneously beyond the information that can be collected from the analysis of diffusion alone. The introduction of electrokinetic forces in nanofluidic devices enables better guiding, confining, releasing, and sorting of a large variety of biological molecules, including proteins, lipid vesicles, and adenoviruses. For instance, by embedding electrodes in a nanochannel, it is possible to realize nanovalves, which are able to tailor the potential energy landscape of nanoobjects using an electric field and guide them from a reservoir with an ensemble of molecules to a confined space.^132^

**Figure 6:**
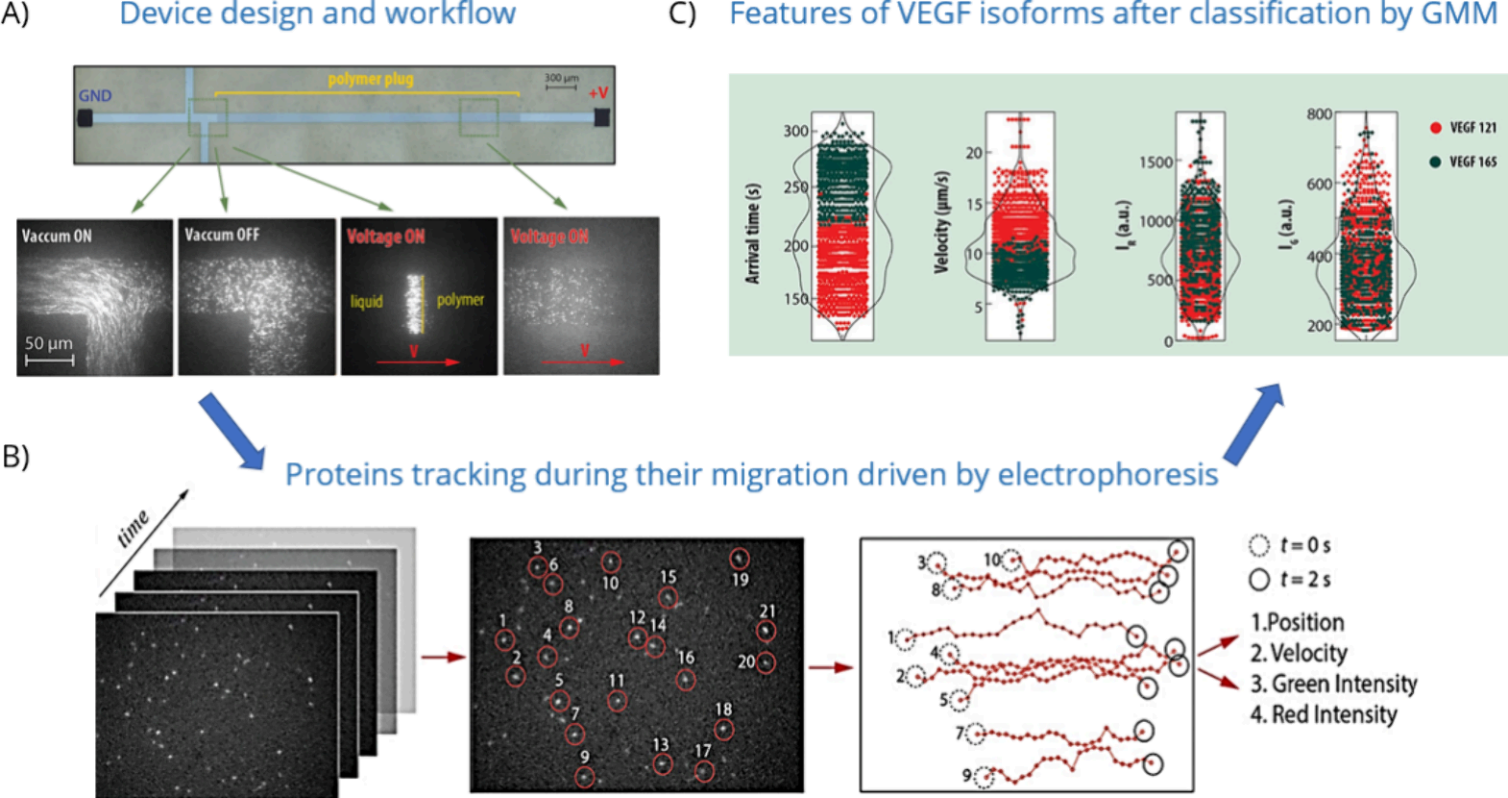
Separation, tracking and identification of individual dually colored proteins. (A) T-shaped nanochannel-based device functionalized with a polyacrylamide gel to slow down the protein motion. Proteins are loaded into the device by applying negative pressure, and their migration is driven toward the gel by electrophoresis. (B) Each movie recorded with an EM-CCD camera consists of a sequence of frames that contain the information on the proteins motion along the channel. First, in each frame the spots corresponding to individual labeled proteins are identified, and then the tracks are reconstructed by means of a Kalmann-based algorithm. The features that are extracted from the analysis of the tracks, namely, the initial position, the mean velocity, and the red and FRET intensities, are used to classify proteins from a solution containing a mixture of proteins. (C) Example of the main features extracted from the analysis of the tracks for the case of two dually labeled VEGF isoforms, namely, VEGF 121 and VEGF 165. From the classification of the data by GMM and PCA, the two isoforms show differences in the values of the main features extrapolated with this method. Reproduced from ref (15). Copyright 2024 Wiley.

Recently, it was shown that individual proteins can be visualized during their electrokinetic-driven migration in a nanochannel without the use of antibodies.^133^ Moreover, further improvement of this method permits high-resolution trajectory tracking of each and every protein, enabled by the fact that the nanochannel confinement in the *z*-direction ensures that the proteins remains in the image plane over extremely long distances (∼100 μm).^15^ This approach can be generalized to any type of biomolecule, but one of the first interesting applications is the ability to separate proteins by their *M*_w_ and identify them based on chemo-elective labeling of two distinct *aa*. The device consists of two lateral channels to load the sample by the use of negative pressure and a main channel in which the proteins migrate through the application of an external voltage (Figure 6a). Before the measurement, the main channel is cast in situ with a polyacrylamide-based solution and a certain portion of it is cross-linked through focused UV light exposure. The polyacrylamide gel-filled nanochannel is used to separate the proteins by their *M*_w_, and at the same time the molecule’s confinement in the *z*-direction facilitates single-molecule sensing.^134^ Molecules are then tracked one-by-one in the nanochannels (Figure 6b). In these devices, the populations of individual proteins arrive at the imaging area and form waves over time, since proteins of different *M*_w_ move with different speeds and arrive at a certain position at different times. Due to the dual *aa* chemoselective labeling of proteins with two different fluorescent probes that are excited via two laser lines, additional information is gained by collecting the fluorescent signals. Notably, the method permits photon collection from both direct excitation of each fluorophore and any possible FRET signals that can further report on the proteins’ *aa* sequence. Consequently, even proteins with similar molecular weights, which are not mass-separated by the gel, can be identified. In fact, proteins are classified based on the analysis of the features extracted from the trajectory, which are the arrival time, the mean velocity, and the red and FRET signals, by means of Gaussian mixture model (GMM) and principal component analysis (PCA) applied to the whole data set containing thousands of trajectories (Figure 6c). Importantly, unlike the previous examples of molecule characterization in nanochannels under the diffusion regime, proteins with low *M*_w_ can also be efficiently tracked. In particular, this method has already been applied for the identification and quantification of a panel of cytokines clinically relevant in bacterial and viral infection, as well as for the discrimination of two splice isoforms of vascular endothelial growth factor (VEGF) from human serum samples.

### Probing Other Biomolecules, Including Individual Liposomes, Extracellular Vesicles and Viruses

Apart from protein identification and the investigation of colloidal particles, nanochannel-based platforms have also been used to characterize heterogeneous samples containing extracellular vesicles (EVs) in solution. Unlike other single-molecule imaging techniques for EV profiling, this does not require their capture on a surface, thus eliminating the possibility of biasing the EV population based on antibody specificity.^135^ In particular, Friedrich et al. determined the particle concentration in a sample containing cell-derived EVs labeled with a lipophilic dye and probed peptide binding to a synthetic lipid vesicle membrane by dual-color detection imaging of the labeled EVs flowing in a nanochannel-based device.^136^ In another study, by changing the electrolyte concentration of the solution used to fill a nanochannel of 200 nm, the electric double layer thickness was tuned and in turn, exosomes of different sizes in the range 50–150 nm were analyzed and sorted by size.^137^ Similarly, EV separation in nanofluidic devices has been successfully performed in chips supporting micro- and nanopillar arrays, tailoring their gaps with an approach called nanometer-scale lateral displacement.^138,139^ In another recent work, liposomes with complementary DNA probes and labeled with either FRET donor or acceptor fluorophores are introduced sequentially and trapped in a funnel-shaped nanochannel to investigate DNA-mediated liposome fusion by collecting the FRET signal from the liposome pairs over time.^140^ Additionally, nanofluidic channels, integrating nanoscaled mixers, filters, and reactors for sample preparation, have been used for the detection and characterization of individual viruses.^141^ Planar nanochannels that consist of two adjoining segments of different heights have enabled to trap and detect different types of labeled capsids, including Herpes simplex virus 1 capsids and hepatitis B virus capsids.^142^ Nanochannels have also been combined with optical interferometry to probe individual viruses by collecting the elastically scattered light generated by a virus as it traverses a laser focus in a process called heterodyne interferometric detection mechanism.^143,144^ Basically, a laser with frequency ω is reflected off a beam splitter and focused into the nanofluidic channel; then, the scattered light is superimposed to a reference beam with frequency ω + Δω and directed into a differential detector. This method has been implemented to recover the size distribution for a mixture of HIV and Sindbis viruses.

Another technique for label-free tracking of single molecules and nanoparticles is based on a single-mode, step-index optical fiber with a nanochannel inside the high-index core.^145^ When the light is guided through the fiber’s core, the optical mode in the fiber overlaps with the nanochannel cross-section, since its diameter is smaller than the wavelength of light. The nanochannel is filled with a liquid solution containing the particles of interest, which act as scattering centers for guided light as they diffuse in the nanochannel. The scattered light travels through the transparent fiber’s cladding and is collected with an objective, which is placed in the orthogonal direction with respect to the fiber axis to enhance the signal-to-background ratio. With a frame rate of 3.5 kHz, nanoparticles as small as 20 nm were tracked for tens of seconds as well as single chlorotic mottle virus (CCMV) virions.

## Conclusions

With the rapid progress of nanofabrication techniques, nanofluidic devices have emerged as powerful tools for biomolecule analyses in ultrasmall volumes. As in the case of microfluidics, complex operations including biomolecule separation, filtration, and mixing are performed on a network of nanochannels, enabling the translation, and in principle the future automation, of samples preparation from the bench to chips. In addition, nanofluidic-based devices have been used for the manipulation of biomolecules, such as the linearization of biopolymers, separation by mass or charge to mass ratios, and their detection at the single-molecule level, which are both beyond the capabilities of microfluidic platforms. We have discussed how, by taking advantage of the physical confinement offered by nanochannels in one or two dimensions, DNA molecules can be completely linearized. This has been exploited to develop optical genome mapping in combination with labeling techniques of specific sequence motifs of DNA and multicolor fluorescence imaging. Nowadays, optical genome mapping together with nanopore-sequencing technologies are the gold standard for genomic studies, both at the clinical level and for the investigation of protein–DNA interactions. Besides optical genome mapping, we have considered the latest applications involving nanofluidic devices, which have received a significant boost in the past few years, as witnessed by the emergence of nanochannel-based platforms capable of protein sizing, mass separation, and discrimination with both multicolor fluorescence imaging and other label-free single-molecule optical techniques. Nanochannels have also been used for the characterization of other individual molecules, such as viruses, liposomes, and EVs. Nevertheless, limitations still exist for biomolecule profiling, including proteoform discrimination, detection of post-translational modifications on proteins, and sizing of EVs with high accuracy. To be developed as the next generation of proteomic and metabolomic sensing platforms, nanochannels still require significant improvements. Further advancements of the fabrication of nanofluidic devices on a large-scale, the automation of sample preparation in ultrasmall volumes directly on-chip, and the optimization of single-molecule optical techniques will be necessary to fully develop nanochannel devices for point-of-care diagnostics. Importantly, nanochannel-based devices are expected to play a prominent role in the following years, as they are compatible with the ultrasmall volumes and concentrations of clinical samples and can play an important role in the upstream analysis and separation of complex mixtures of biomolecules when integrated with other emergent single-molecule technologies, i.e., nanopores. As previously discussed, the use of nanochannels for advanced applications regarding proteins sizing, separation, and identification is rapidly growing, and we envision that these devices will soon show even more powerful capabilities in proteomics, such as proteoform discrimination and the identification of post-translational modifications. In addition, nanochannel-based platforms are compatible with a wide range of different types of biomolecules, including exosomes and lipid vesicles, and we believe that upcoming studies will show high-throughput profiling and characterization with unprecedented sensitivity and resolution compared to the current state-of-the art bulk technologies.
